# Development of Green HPTLC method for simultaneous determination of a promising combination Tamsulosin and Mirabegron: stability-indicating assay was examined

**DOI:** 10.1186/s13065-023-01043-9

**Published:** 2023-09-30

**Authors:** Maha M. Abou El-Alamin, Safaa S. Toubar, Dina A. Mohamed, Marwa I. Helmy

**Affiliations:** Pharmaceutical Analytical Chemistry Department, Faculty of Pharmacy, Helwan University, P. O. Box 11795, Cairo, Egypt

**Keywords:** Green assessment, HPTLC, Mirabegron, Tamsulosin, Stability study

## Abstract

Recently, mirabegron has been added to tamsulosin to treat overactive bladder in men with benign prostatic hypertrophy. A Rapid, selective, sensitive, and green high-performance thin-layer chromatography (HPTLC) approach was developed for the simultaneous determination of tamsulosin (TAM) and mirabegron (MIR) in pure and laboratory-prepared mixture. Complete separation was obtained on silica gel F_254_ using the solvent system methanol-ethyl acetate-ammonia (3:7:0.1, v/v). Short-wave ultraviolet light at 270 nm was used to view the chromatographic bands. For MIR and TAM, the suggested technique revealed compact spots with retention factor R_f_ values of 0.42 and 0.63, respectively. Within concentration ranges of 0.15–7.5 µg/band and 0.05–2.5 µg/band, good linearity was observed, with mean percentage recoveries of 100.04 ± 0.56 and 99.98% ± 0.95 for MIR and TAM, respectively. Green assessment of the developed HPTLC technique was estimated using different green analytical chemistry metrics such as Analytical eco-scale Analytical GREEness (AGREE), and Green Analytical Procedure Index (GAPI) metrics. The proposed method was effectively used as a stability-indicating assay to assess the presence of MIR and TAM in the pharmaceutical dosage form in the presence of their degradation product. The statistical analysis showed high precision and accuracy.

## Introduction

**Fig. 1:**
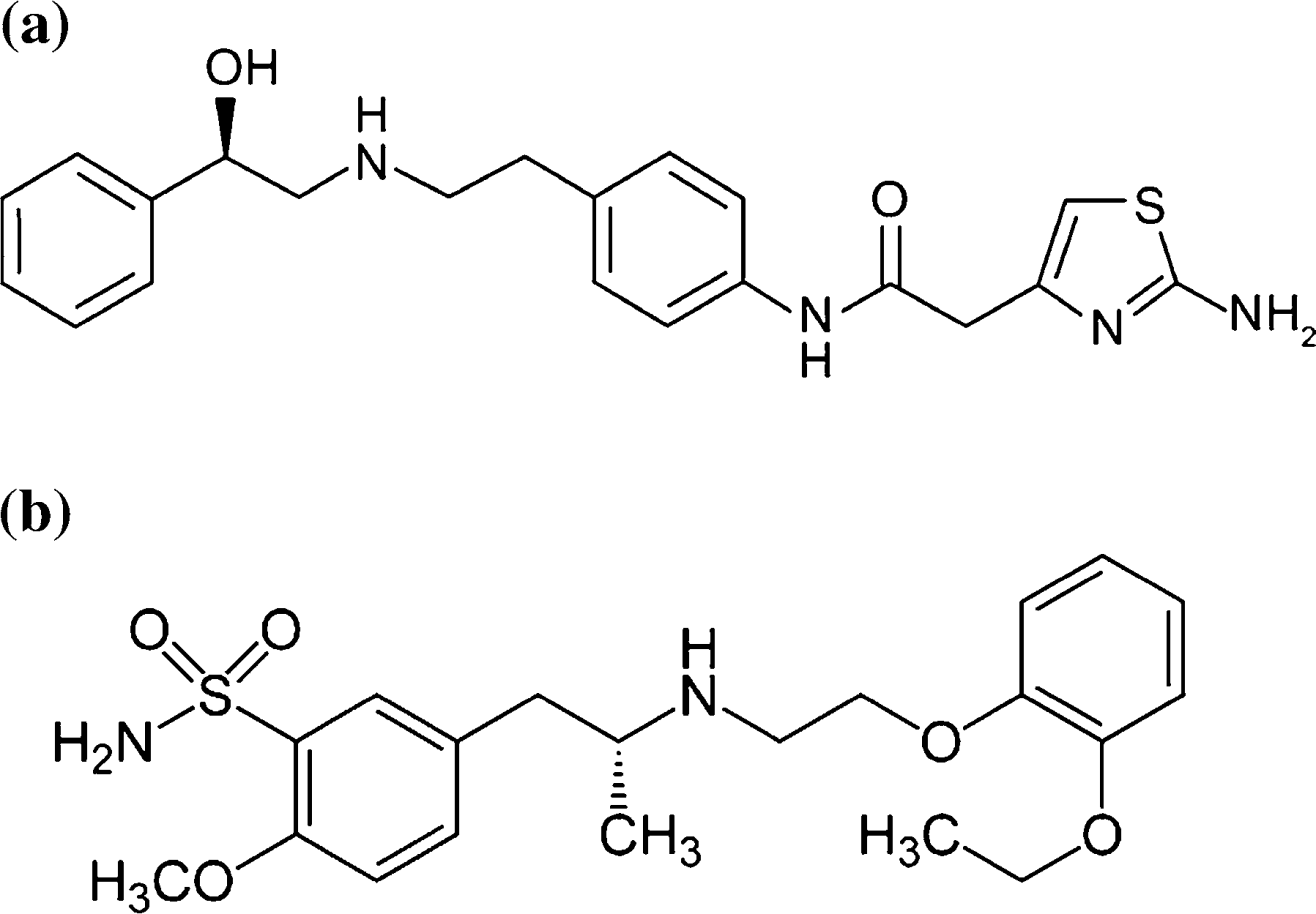
**a** Structure of MIR. **b** Structure of TAM

Mirabegron (MIR) Fig. 1a, is chemically named as [2-(2-amino-1,3-thiazol-4-yl)-N-[4-(2-{[(2R)-2- hydroxy-2-phenylethyl]amino}ethyl)phenyl]acetamide]. MIR is the first 3-adrenoreceptor agonist approved in the treatment of overactive bladder (OAB) as an alternate to antimuscarinic agent [1]. It relaxes the detrusor smooth muscles in the urine bladder, increasing bladder capacity. It is a selective activator of beta-3 adrenergic receptors. The literature reviews showed different analytical methods for the determination of MIR in bulk, pharmaceutical dosage form, and biological fluids, such as spectrophotometric [2–7], spectrofluorometric [3], LC–MS/MS [8–11], HPLC [12–16] HPTLC [17–20], and electrochemical methods [21].

Tamsulosin HCl (TAM), Fig. 1b, is 5-[(2R)-2-[[2-(2-Ethoxyphenoxy) ethyl] amino] propyl]-2-methoxybenzenesulfonamide hydrochloride. It is a α1-adrenoceptor (AR) antagonist. It is used in treatment benign prostatic hyperplasia urinary symptoms and to control renal calculi by decreasing ureteric smooth muscle contraction [22]. According to the literature reviews, there are numerous analytical approaches for analyzing TAM, which include spectrophotometric [23–26], voltammetric [27, 28], HPTLC [29–38], HPLC [5, 36, 38–53], and LC–MS/MS [43, 54–59] methods.

Nowadays, MIR is added to TAM in treating overactive bladder in men with benign prostatic hypertrophy as it improves overactive bladder symptoms and decreases micturition frequency [60]. This necessitates the development of a simple, specific technique for determining TAM and MIR in pure and pharmaceutical dosage forms. The literature review revealed that no analytical methods were reported for the simultaneous determination of the cited drugs.

Literature review showed that HPLC, HPTLC, LC–MS/MS techniques are sensitive, precise, and accurate for the determination of TAM or MIR. No method has been reported for the simultaneous determination of MIR and TAM. According to green assessment, HPTLC has advantages over other techniques as being fast, using a few microliters of an organic solvent and a few micrograms of solute to examine and quantify a target analyte. Different HPTLC methods have been reported for the determination of tamsulosin in combination with various drugs, only one HPTLC method has also been reported for the determination of mirabegron with solifenacin succinate. The simultaneous determination of TAM and MIR is challenging as MIR dosage is 50 mg and TAM is 0.4 mg. Thus, the aim of this study was to conduct a sensitive, accurate and precise HPTLC method for the separation and quantitation of MIR and TAM in bulk and their laboratory-prepared mixture.

High-performance thin-layer chromatography (HPTLC) has emerged as a major step toward improved separation quality, with smaller silica particles resulting in faster analysis, sharper peaks, improved resolution, and increased sensitivity. Furthermore, HPTLC is a micro-scale technique that only requires a few microliters of a volatile solvent and a few micrograms of solute to examine and quantify a target analyte [61].

Green chemistry is described as "the use of chemistry techniques and approaches to limit or eliminate the use or creation of reagents, solvents, and byproducts that are harmful to human health or the environment." As a result, reducing or avoiding the dangers associated with processes and products is an important part of green chemistry. The twelve principles of green analytical chemistry (GAC) serve as a guideline for achieving greenness in analytical procedures [62]. Various metrics for evaluating the greenness of analytical techniques have been established. Some are limited to specific analytical procedures, while others are more common and can be used for a wide range of operations [63]. The developed HPTLC technique was evaluated using different metrics, including, Analytical Eco-Scale [64], Green analytical procedure index (GAPI) [65], and Analytical GREEness (AGREE) [66] metrics.

Thus, this work will focus on developing a green, rapid, and selective HPTLC method for simultaneously determining the cited drugs in the pure and laboratory-prepared mixture. Also, a stability-indicating assay has been applied to the suggested HPTLC technique to determine MIR and TAM in their pharmaceutical dosage form without interference with their degradation products.

## Experimental

### Instrumentation and chromatographic conditions

TLC silica gel 60 F_254_ aluminum sheets plates 20 × 20 cm with 0.25 mm thickness were used to develop the HPTLC approach (E. Merck, Darmstadt, Germany). CAMAG autosampler was used to detect samples in the form of discrete bands (Muttenz, Switzerland). TLC Scanner 3 model S/N 230495 was used throughout the experiments. The scanning speed (20 mm/s) and slit dimension (6.00 × 0.45 mm) were used. For 30 min, plates were developed in a 20 cm × 10 cm twin-trough glass chamber saturated with the appropriate mobile phase of methanol‒ethyl acetate-ammonia (3:7:0.1, v/v). With a progress time of 15 min, the plates were developed to a distance of 75 mm. The plates were dried for 2 min at room temperature before being analyzed at 270 nm with the absorbance mode utilizing a deuterium lamp as the radiation source. The densitometric study was performed using WinCATS software.

### Materials and methods

#### Pure standard

Mirabegron (MIR) (purity 99.98%, Apex pharma, Egypt). Tamsulosin (TAM) (purity 100.31%, Macryl, Cairo, Egypt).

#### Pharmaceutical dosage form

Bladogra® 50 mg MIR per tablet (batch No. MT 8701019, Apex Pharma, Cairo, Egypt). Tamsulosin® 0.4 mg TAM per capsule (batch No. 2023970, Marcryl, Cairo, Egypt). Both preparations were obtained from the local market.

#### Chemicals and reagents

Methanol and ethyl acetate (Fisher chemical, United States), Sodium hydroxide, hydrochloric acid, hydrogen peroxide, and ammonia (El-Nasr company, Cairo, Egypt), All of the studies were carried out with double-distilled water.

### Standard solution

- Stock standard solution: 1 mg/mL was prepared by transferring 10.0 mg of MIR or TAM separately into 10-mL volumetric flasks, dissolved in methanol, and completed to the mark with the same solvent.
- Working solution mixture: Aliquots of 7.5 mL and 2.5 mL from the stock solutions of MIR and TAM were transferred into a 10-mL volumetric flask to give a final concentration of 0.75 mg/mL MIR and 0.25 mg/mL TAM.

### Construction of the calibration graph and analysis of pure bulk powders

Aliquots of stock solution (0.2–10.0 µL) from the working solution were applied in triplicate on a TLC plate using CAMAG Linomat autosampler with CAMAG micro syringe. The plates were developed using the optimized mobile phase and scanned as under Section chromatographic conditions. The average peak areas were plotted against the corresponding concentrations ranging from (0.15–7.5 µg/band) and (0.05–2.5 µg/band) for MIR and TAM, respectively to get the calibration graph. The regression equations were constructed.

### Analytical application

#### Determination of MIR and TAM in their laboratory-prepared mixture

Laboratory mixture was prepared in the same ratio as their tablet doses. Five tablets of Bladogra® 50 mg were finely powdered and an accurate amount equivalent to 50 mg of the drug was transferred into a 100-mL volumetric flask. Five Tamsulosin® 0.4mg capsules were weighed individually after evacuating the capsules then the contents of the five capsules were mixed well and the amount of the powder equivalent to 0.4 mg of TAM was weighed, transferred into the same flask containing MIR with 70 mL of methanol, and sonicated for 30 min, the solution was completed with methanol and filtered through a 0.45 μm filter. From this solution 12.5, 14.0, and 15.0 µL were spotted on the plate. The spot was developed using the mobile phase stated under the chromatographic conditions.

#### Forced degradation studies

According to ICH guidelines, the aim of stability testing is to provide indication on how the quality of a drug substance or drug product differs with time under different conditions.

*Solution A:* Five Bladogra® 50 mg tablets were finely ground, and an exact amount equal to 10 mg was put into a 10 mL volumetric flask with 8 mL of methanol, sonicated for 30 min, complete with the same solvent, and filtered through a 0.45 m filter.

*Solution B:* Thirty capsules of Tamsulosin® capsules were weighed and completed as described above.

##### Alkaline degradation

Two mL of solutions A and B were transferred separately with 2 mL of 2N NaOH into a rounded bottom flask and heated under reflux in a water bath at 80 °C for 3h. The solutions were neutralized using 2N HCl. The resultant solutions were transferred separately to a 10-mL volumetric flask and completed to the mark with methanol. Finally, an aliquot of 10 µL of MIR and 3.5 µL TAM were spotted separately in triplicate on the TLC plate.

##### Acidic degradation

Two mL of solutions A and B were transferred separately with 2 mL of 2N HCl into rounded bottom flask and heated under reflux in a water bath at 80 °C for 3h. The solution was neutralized using 2N NaOH, and proceeded as under alkaline degradation.

##### Oxidative degradation

Two ml of solutions A and B with 2 mL of 30% H_2_O_2_ were transferred separately into a rounded bottom flask and left at room temperature for 3 h, then, the mixture was boiled in the water bath to expel excess H_2_O_2_ and proceeded as above.

##### Thermal degradation

Two mL of solutions A and B were transferred separately with 2 mL water into a rounded bottom flask and heated under reflux in a water bath at 80 °C for 24 h; then the solution was cooled and proceeded as above.

##### Photolytic degradation

Two mL of solutions A and B were transferred separately into 10-ml volumetric flask, left in daylight for 3 days, and 1 day for MIR and TAM, respectively and proceeded as above.

## Results

The suggested technique was applied efficiently as a stability-indicating HPTLC technique to determine MIR and TAM alone or in combination as the degradation products R_f_ away from the spot of the intact drug. Also, it could be used for analysis in pharmaceutical industries as it gives a short time of analysis. Moreover, it could be used for the quantitative analysis of MIR and TAM in their dosage form with an excellent percentage recovery.

### Method development and optimization

It was important to check the effects of several variables in order to optimize the chromatographic conditions.

#### Appropriate wavelength selection

**Fig. 2:**
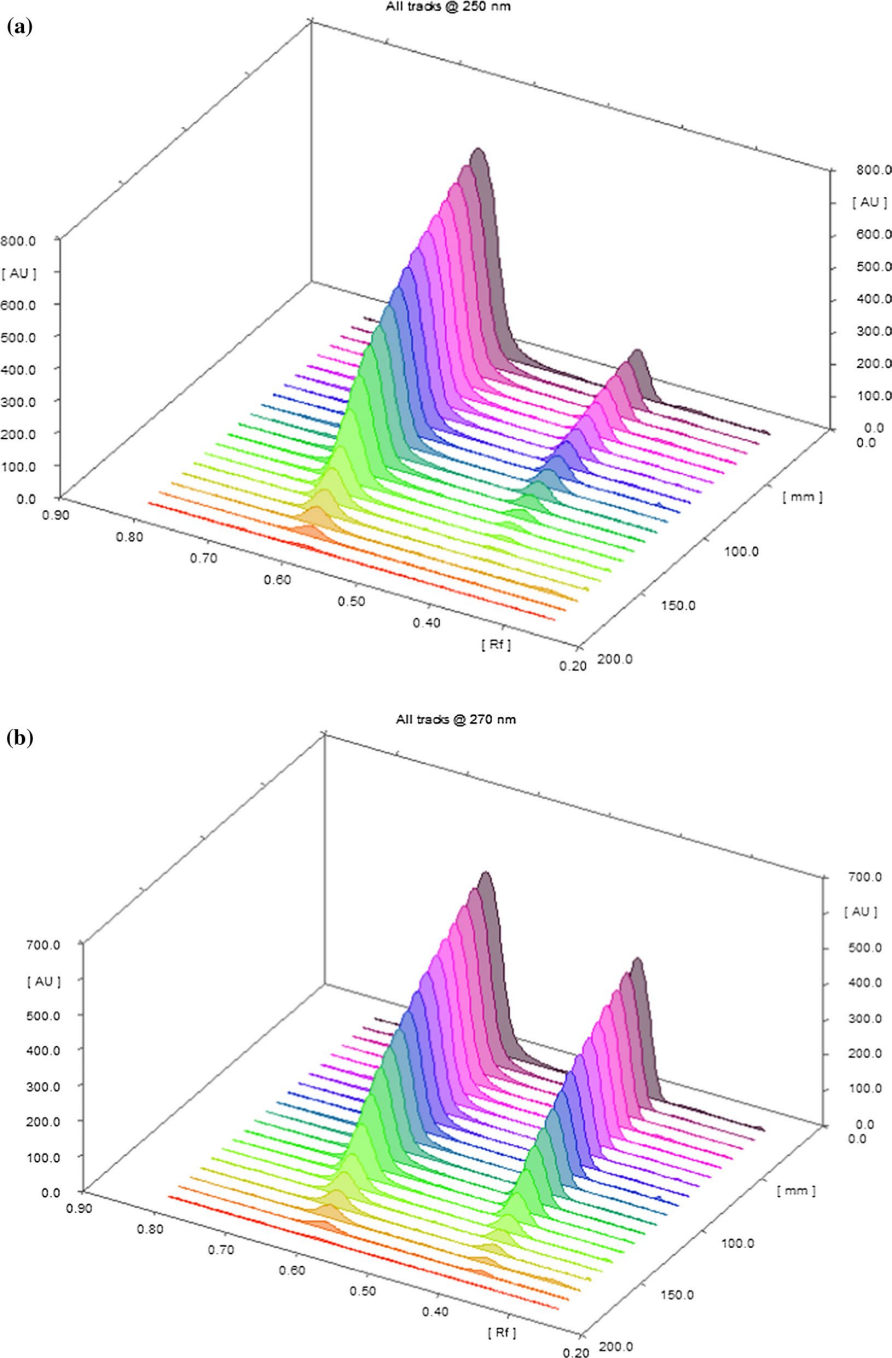
3D densitogram of MIR and TAM at **a** 250 nm, **b** 270 nm

After experimenting with several scanning wavelengths, as shown in Fig. 2a, b below at 250 nm, the absorbance of mirabegron was dramatically decreased, 270 nm provided the optimum sensitivity for quantifying and identifying MIR and TAM.

#### Choice of appropriate mobile phase

**Fig. 3:**
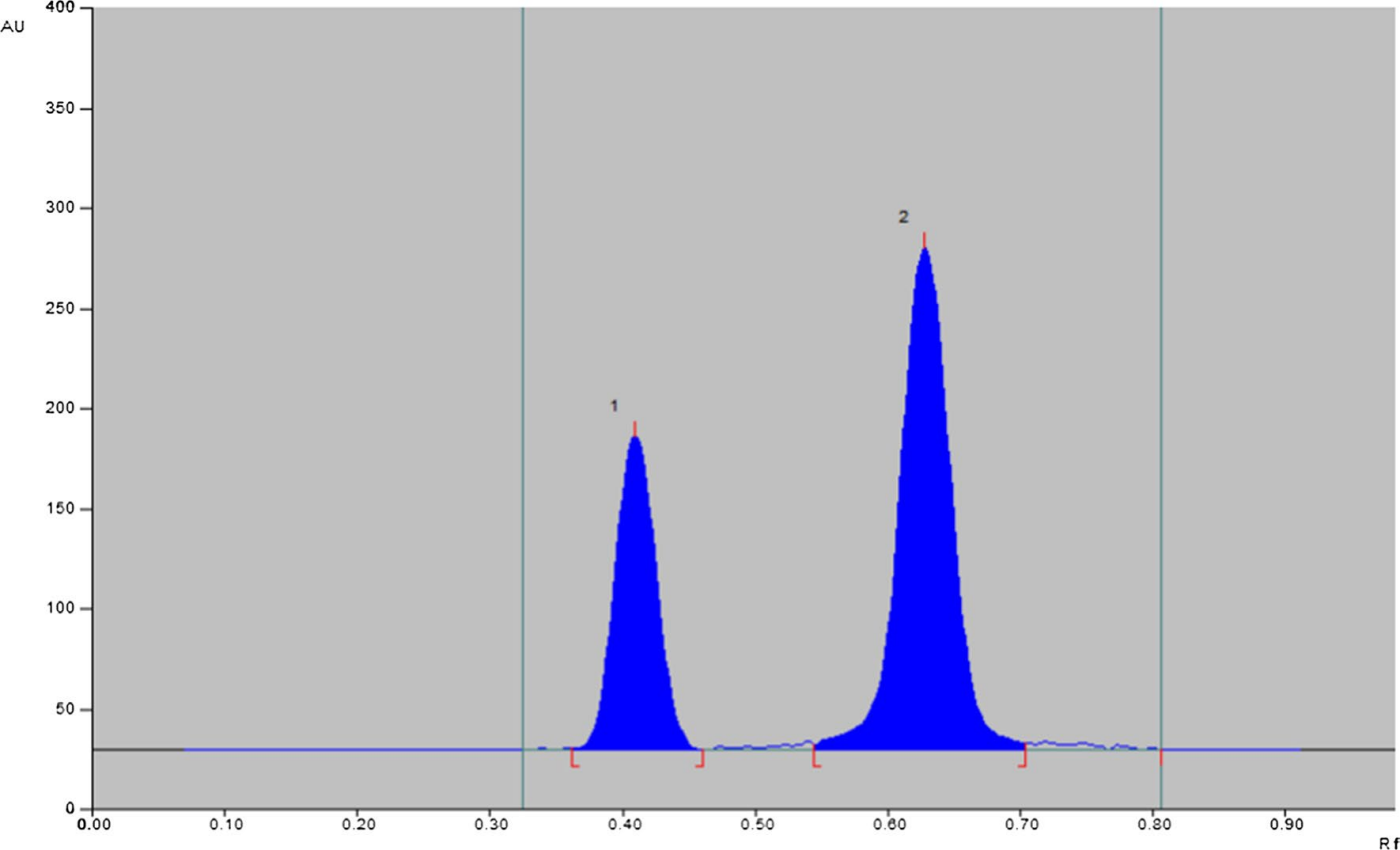
Thin-layer chromatogram of (1) MIR and (2) TAM on silica gel plate F254 using methanol‒ethyl acetate‒ammonia (3:7:0.1, V/V)

**Fig. 4:**
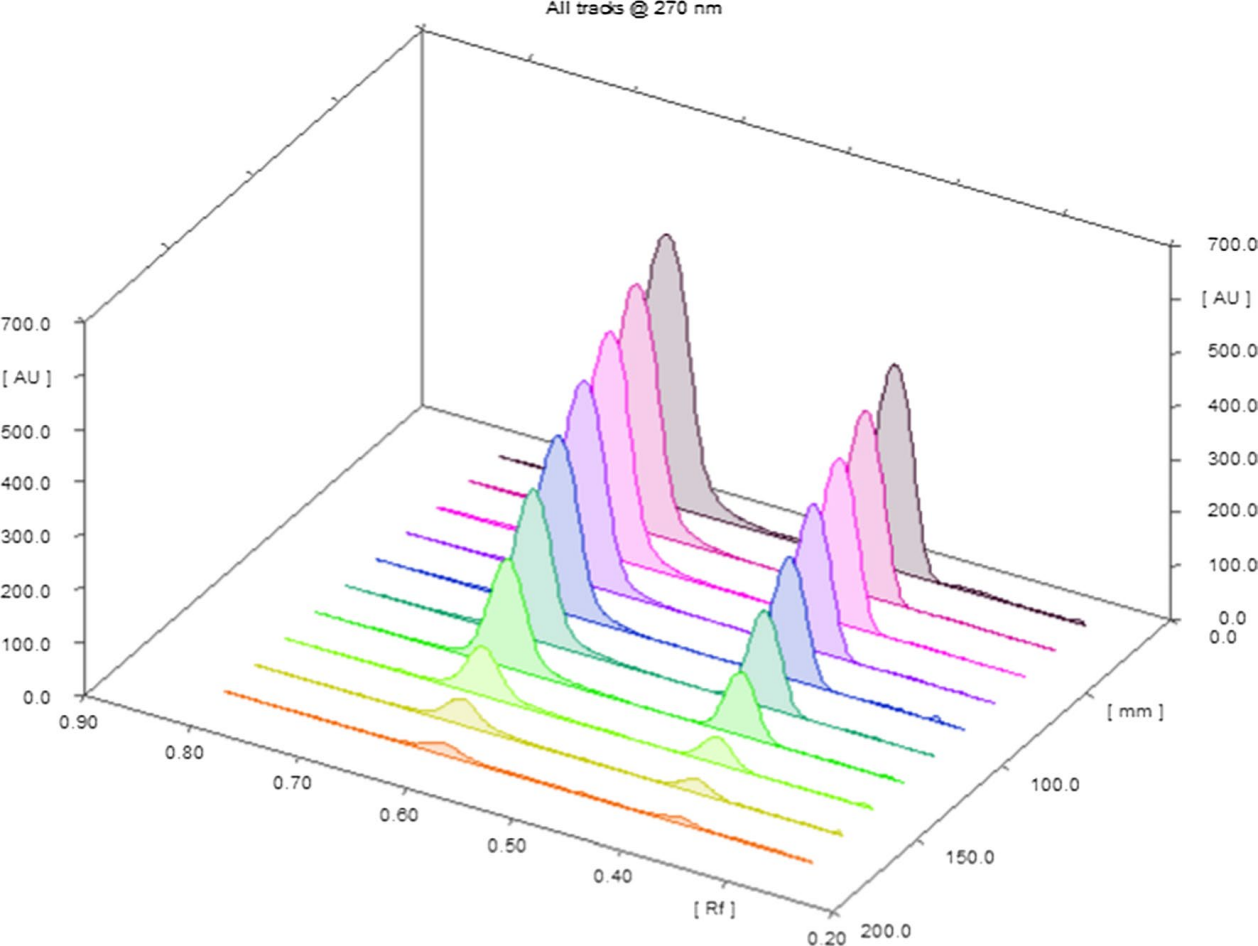
3D densitogram of MIR and TAM on silica gel 60 F254 TLC plate

Before carrying out quantitative HPTLC analysis, a qualitative study on a silica gel plate (5 × 10 cm) was carried out to choose the optimum mobile phase for the simultaneous determination of TAM and MIR. Visualization of the spot was carried out using iodine vapors. Different compositions and ratios of the mobile phase were studied for separation. Different mobile phase was examined depending upon pH of the system and pka of the drugs. Knowing that pka of mirabegron 4.5, 8.5 and pka of tamsulosin 9.28, 9.93, the pH was adjusted using ammonia. Ethyl acetate and methanol are commonly used as solvents in HPTLC, Ethyl acetate and methanol have different polarity and solubility properties, which affect the migration and separation of the compounds on the TLC plate. Ethyl acetate can dissolve non-polar compounds, while methanol is a polar solvent that can dissolve polar compounds. Methanol interact with hydroxyl, amine and sulfonamide groups of tamsulosin and also with hydroxyl, amine and amide groups of mirabegron giving well separation of two drugs [67–73]. A higher portion of ethyl acetate will result in a faster and more efficient elution of tamsulosin. Ammonia act as a modifier that can adjust the polarity and pH of the mobile phase in chromatography. Ammonia interact with polar groups in the TAM and MIR and enhance their solubility in the mobile phase. Ammonia improve the resolution of TAM and MIR and prevent tailings of the cited compounds. Different ratios of ethyl acetate to methanol and ammonia were examined to obtain the optimum separation of bands of the two cited drugs. Well separation with good resolution and sharp symmetrical peaks was achieved by using methanol-ethyl acetate-ammonia (3:7:0.1, v/v). It was found that methanol-ethyl acetate-ammonia (3:7:0.1 V/V) was the optimum mobile phase for separating MIR and TAM with R_f_ values of 0.42 and 0.63 for MIR and TAM, respectively, as shown in Figs. 3,4.

#### Slit dimensions of scanning light beam

The slit dimensions of the scanning laser beam should completely cover the scanned track's band dimensions, with no interference from the opposite track. The ideal slit size with the best sensitivity was found to be (6.00 × 0.45 mm).

#### Effect of chamber saturation time

The recommended chamber saturation time for conventional TLC ranges from 15 to 30 min. Different saturation time was studied 15, 20, 30 min. The optimum resolution was achieved at 30 min.

#### Effect of time from development to scanning

The solvent on the plate can evaporate over time, which can change the concentration and distribution of the components on the plate. This can affect the peak shape, peak area, and resolution of the components. Therefore, it is recommended to scan the plate as soon as possible after development to avoid solvent evaporation. 2 min was enough for drying of the plate in our experiment and there is no difference between peak shape when varying this time from 2 up to 6 min.

### Method validation

The suggested technique was validated according to (ICH) guidelines Q2 (R1) [74]. The results are shown in Table 1.

**Table 1:** Regression data for the calibration curve using silica gel 60 f254 TLC plate

	MIR	TAM
Range (µg/band)	0.15–7.50	0.05–2.50
Slope	1344.20	5167.60
Intercept	1331.70	416.47
Square correlation coefficient (R2)	0.9999	0.9994
%Recovery ± SD	100.04 ± 0.56	99.98 ± 0.95
%RSD	0.559	0.947
LOD (µg/band)	0.09	0.03
LOQ (µg/band)	0.15	0.05

#### Linearity and range

**Fig. 5:**
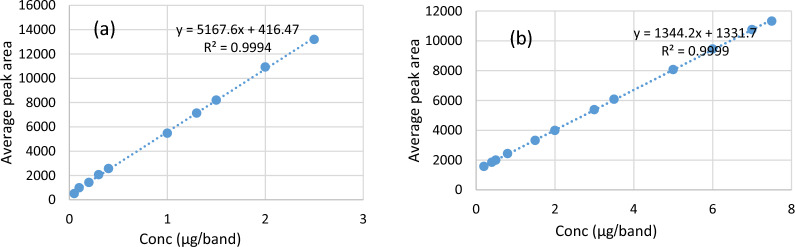
**a** Calibration curve of TAM, **b** calibration curve of MIR

From the regression plot, as shown in Fig. 5a, b, good linearity was achieved within the concentration ranges of (0.15–7.5), (0.05–2.5) µg/band for MIR and TAM, respectively. Linearity data is illustrated in Table 1.

#### Limit of detection (LOD) and limit of quantification (LOQ)

LOD and LOQ were examined experimentally based on visual inspection [75].

##### Detection limit

Based on Visual Evaluation Visual evaluation may be used for non-instrumental methods but may also be used with instrumental methods. The detection limit is determined by the analysis of samples with known concentrations of analyte and by establishing the minimum level at which the analyte can be reliably detected.

By spotting different concentration of solutions. LOD was found to be 0.09, 0.03 µg/band for of MIR and TAM, respectively.

##### Quantitation limit:

Based on Visual Evaluation Visual evaluation may be used for non-instrumental methods but may also be used with instrumental methods. The quantitation limit is generally determined by the analysis of samples with known concentrations of analyte and by establishing the minimum level at which the analyte can be quantified with acceptable accuracy and precision.

LOQ was found to be 0.05, 0.15 µg/band for of MIR and TAM, respectively.

The results are illustrated in Table 1.

#### Accuracy

Accuracy of the proposed method was found to be 100.57 ± 0.52 and 100.35 ± 0.53 for standard MIR and TAM, respectively, as shown in Table 2 and 99.69 ± 0.68 and 99.87 ± 0.9 for MIR and TAM, respectively, in their laboratory prepared binary mixture as shown in Table 3. The accuracy of our proposed method was also proved by statically comparing it with the reported HPLC method. Student's t-test and F-test showed no significant difference between the proposed and reported methods, as shown in Table 4.

**Table 2:** Determination of MIR and TAM in combined standard solution

MIR		TAM	
Taken (µg/band)	Found (%)^a^	Taken (µg/band)	Found (%)^a^
0.75	101.13	0.25	100.49
3.75	100.49	1.25	100.79
7.50	100.09	2.50	99.77
Mean ± SD	100.57 ± 0.52		100.35 ± 0.53

^a^Each result is the average of three separate determinations

**Table 3:** Determination of MIR and TAM in their laboratory-prepared binary mixture

MIR		TAM	
Taken (µg/band)	Found (%)^a^	Taken (µg/band)	Found (%)^a^
6.25	99.27	0.05	99.04
7.00	99.14	0.056	99.73
7.50	100.65	0.06	100.83
Mean ± SD	99.69 ± 0.68		99.87 ± 0.90

^a^Each result is the average of three separate determinations

**Table 4:** Statistical comparison between the results obtained by suggested HPTLC and the reported HPLC method

Parameters	HPTLC		Reported method	
Drugs	MIR	TAM	MIR^a^ [16]	TAM^b^ [46]
Mean ± SD	99.69 ± 0.68	99.87 ± 0.9	100.4 ± 0.25	100.4 ± 0.45
N	3	3	3	3
Student’s t-test (2.776)^c^	0.25	0.4		
F-test (19)^c^	7.39	4		

^a^HPLC using C18, mobile phase acetonitrile–potassium dihydrophosphate 40:60 (v/v) at 249 nm

^b^HPLC using C8, mobile phase acetonitrile–potassium dihydrophosphate 45:55 (v/v) at 240 nm

^c^The tabulated values of t and F at p = 0.05

#### Precision

Intra and inter-day precisions were examined. The results are shown in Table 5**,** indicating the proposed method's high precision.

**Table 5:** Intra-day and inter-day precisions for the determination of MIR and TAM in combined standard solution

Drug name	MIR			TAM		
Conc. µg/band	0.75	3.75	7.5	0.25	1.25	2.5
Intra-day precision ± SD	99.9 ± 0.37	99.1 ± 0.12	100.2 ± 0.075	99.29 ± 0.39	99.7 ± 0.14	100.07 ± 0.14
Inter-day precision ± SD	100.35 ± 0.73	99.46 ± 0.2622	100.36 ± 0.14	98.91 ± 0.13	99.1 ± 0.3	99.8 ± 0.11

#### Robustness

The robustness of the suggested technique is determined by its ability to stay unaffected by a minor change that may occur during the analytical procedures. Changing the mobile phase ± 0.1 mL composition for methanol and ethyl acetate and ± 0.01 for ammonia had no effect on the retention factor of the cited drugs as shown in Table 6.

**Table 6:** Robustness result of the proposed method by changing volume of components mobile phase

Mobile phase	Methanol			Ethyl acetate			Ammonia		
mls	2.9	3	0.1	6.9	7	7.1	0.09	0.1	0.11
% Recovery	98.34	100.10	98.18	100.62	100.10	99.25	97.85	98.18	98.86
%mean recovery	98.22			99.99			98.29		
± SD	0.56			0.56			0.42		
% RSD	0.57			0.56			0.43		

#### Selectivity

The ability of the suggested method to detect medicines in a laboratory-prepared binary mixture without interference from common excipients was investigated. Forced degradation tests were used to assess the validity and specificity of the proposed method for studying medication stability. The results presented in section "Results of the stability studies" demonstrated that the suggested technique is selective and capable of determining MIR and TAM in the presence of their degradation products.

#### Specificity

**Fig. 6:**
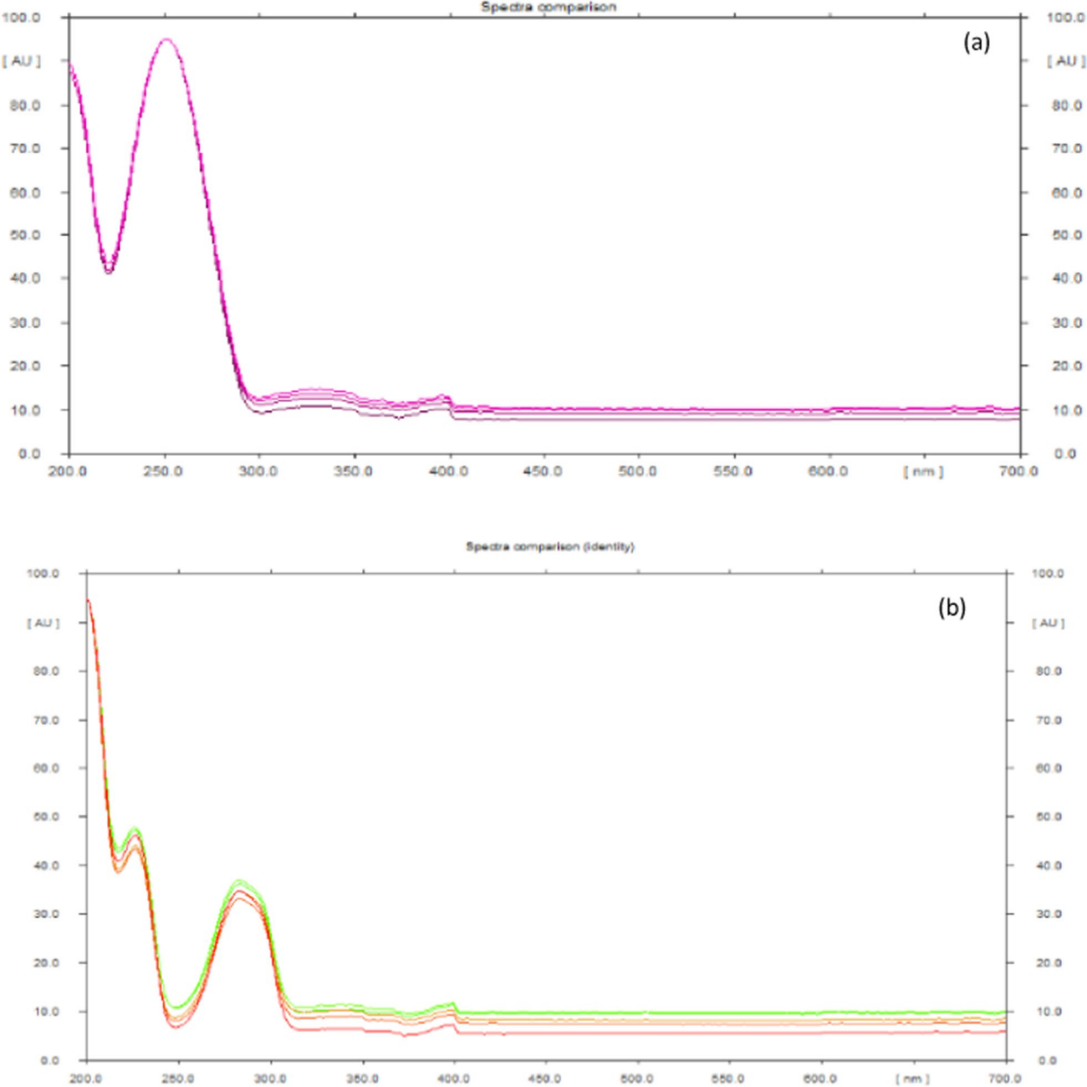
Superimposed UV spectra of standard and dosage form **a** MIR, **b** TAM

The specificity of the suggested technique was evaluated through spectral comparison of the peak purity using winCATS® as shown in Fig. 6a, b for MIR and TAM, respectively. The peak purity was evaluated at three levels: peak start, apex, and end.

### Application to the pharmaceutical formulation

The suggested technique was effectively applied for the determination of MIR and TAM in their laboratory prepared mixture (Table 3).

#### Simultaneous determination of TAM and MIR in their laboratory prepared binary mixture

TAM and MIR in their laboratory-prepared combination were quantified using a standard addition procedure. TAM and MIR reference standards were weighed and added separately to a laboratory-prepared TAM and MIR mixture at concentration levels of 75, 100, and 150% for TAM and at 25, 50, and 75% for MIR. Each sample was prepared in triplicate. The results in Table 7 shown a high accuracy of the proposed method.

**Table 7:** Standard addition technique of simultaneous determination of MIR and TAM in the laboratory prepared mixture

Drug	Amount taken (ng/band)	Pure added (ng/band)	Pure found (ng/band)	% Found
MIR	4000.00	1000.00	996.90	99.69
		2000.00	2002.10	100.10
		3000.00	2979.80	99.33
Mean				99.71
%RSD				0.39
TAM	32.00	24.00	23.98	99.92
		32.00	32.13	100.40
		48.00	48.18	100.37
Mean				100.23
%RSD				0.27

#### Results of the stability studies

The suggested technique was applied effectively to determine the stability of both MIR and TAM in their dosage form under various stress conditions: alkaline, acidic, oxidative, thermal, and photolytic conditions. The developed method was applied effectively as a stability-indicating HPTLC method to determine of drugs alone or in combination as the degradation products R_f_ away from the spot of the intact drugs.

##### Alkaline conditions

**Fig. 7:**
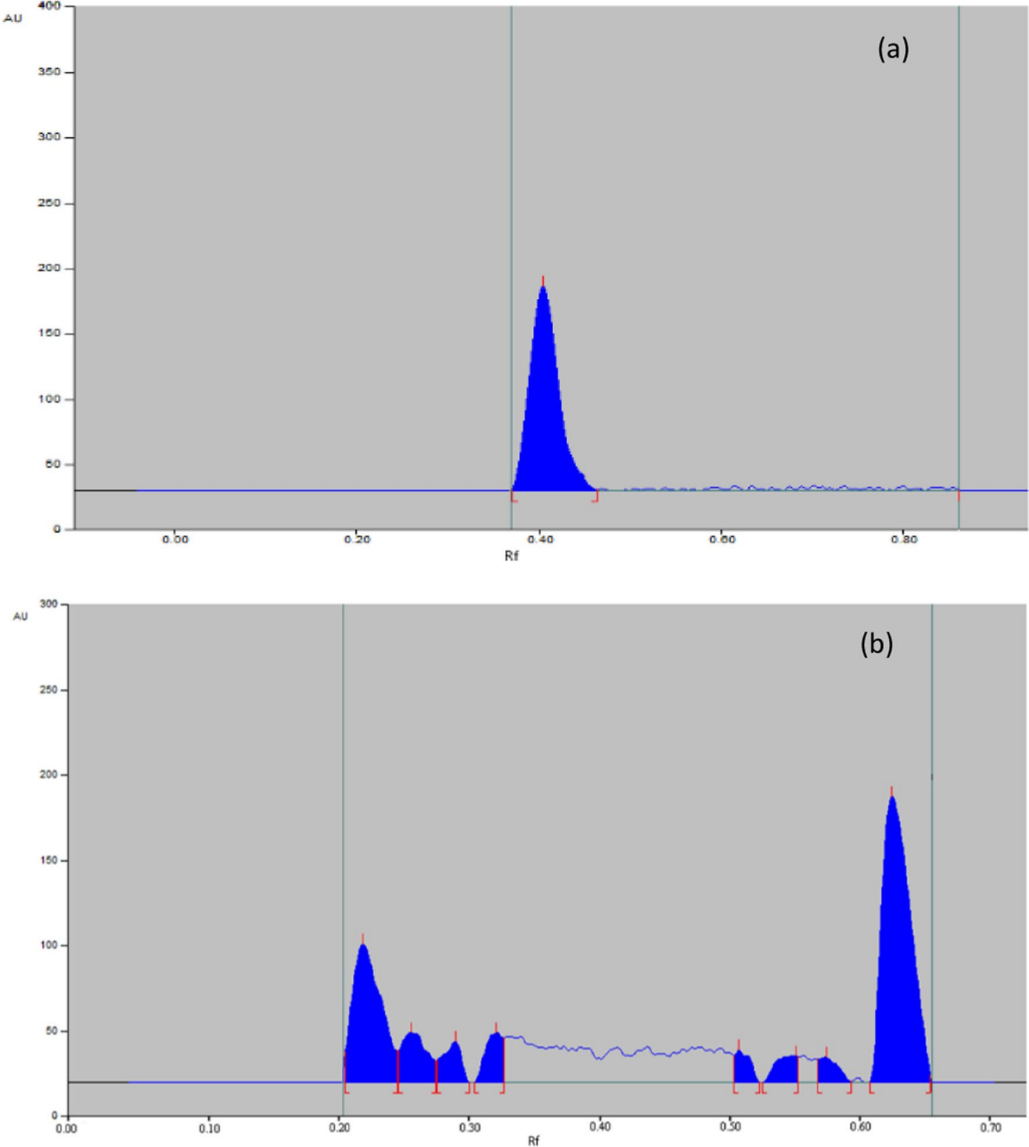
Representative chromatogram obtained the following exposure of MIR (**a**) and TAM (**b**) in their pharmaceutical dosage form to alkaline degradation

Both MIR and TAM were found to be liable to alkaline hydrolysis, 90% of MIR was recovered with no additional peak as shown in Fig. 7a, while 63% of TAM was recovered with additional peaks as shown in Fig. 7b.

##### Acidic conditions

**Fig. 8:**
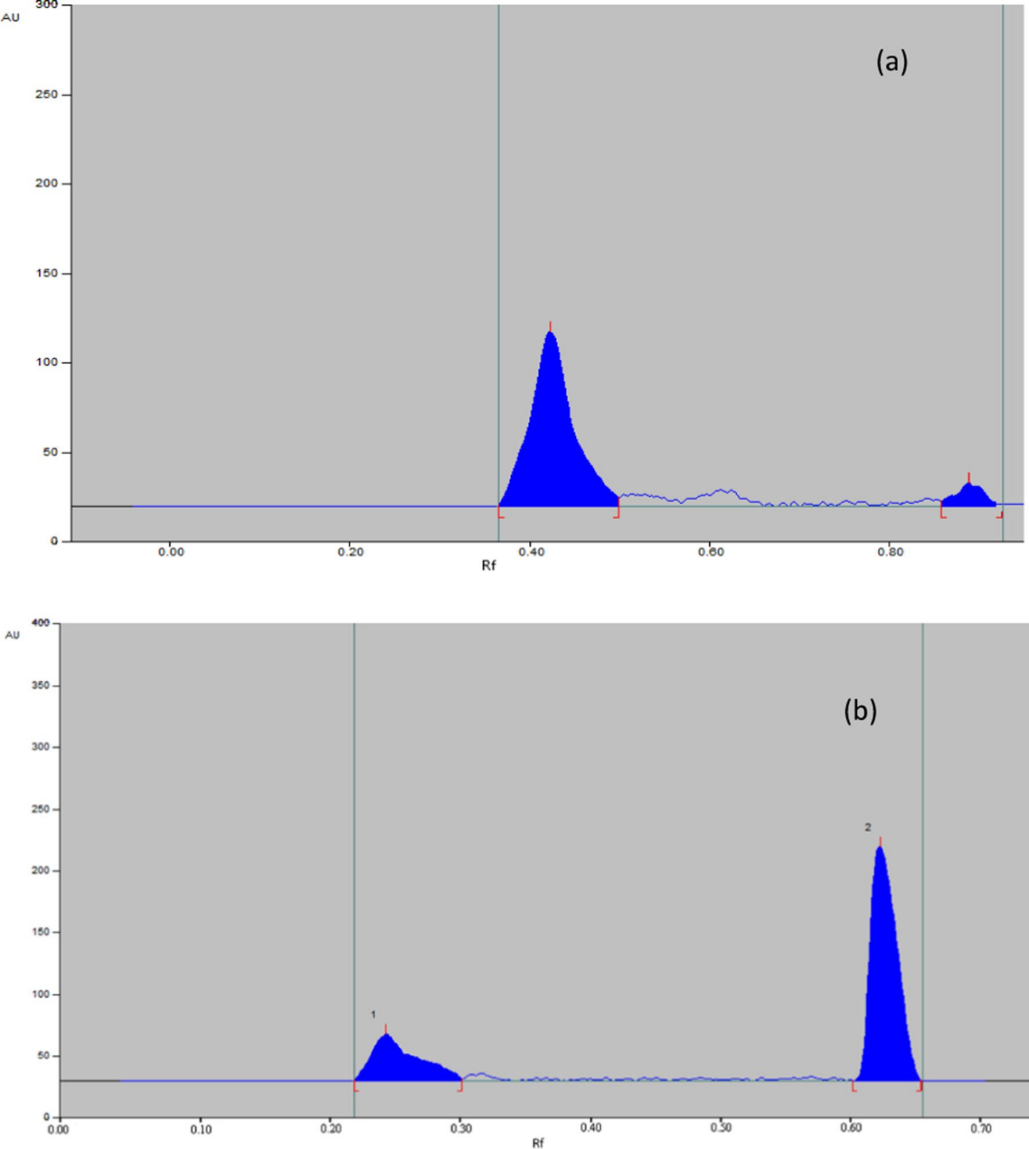
Representative chromatogram obtained the following exposure of MIR (**a**) and TAM (**b**) in their pharmaceutical dosage form to acidic degradation

Both MIR and TAM were found to be liable to acidic hydrolysis. 88% of MIR was recovered with an additional peak, as shown in Fig. 8a, while 64% of TAM was recovered with an additional peak, as shown in Fig. 8b.

##### Oxidative conditions

**Fig. 9:**
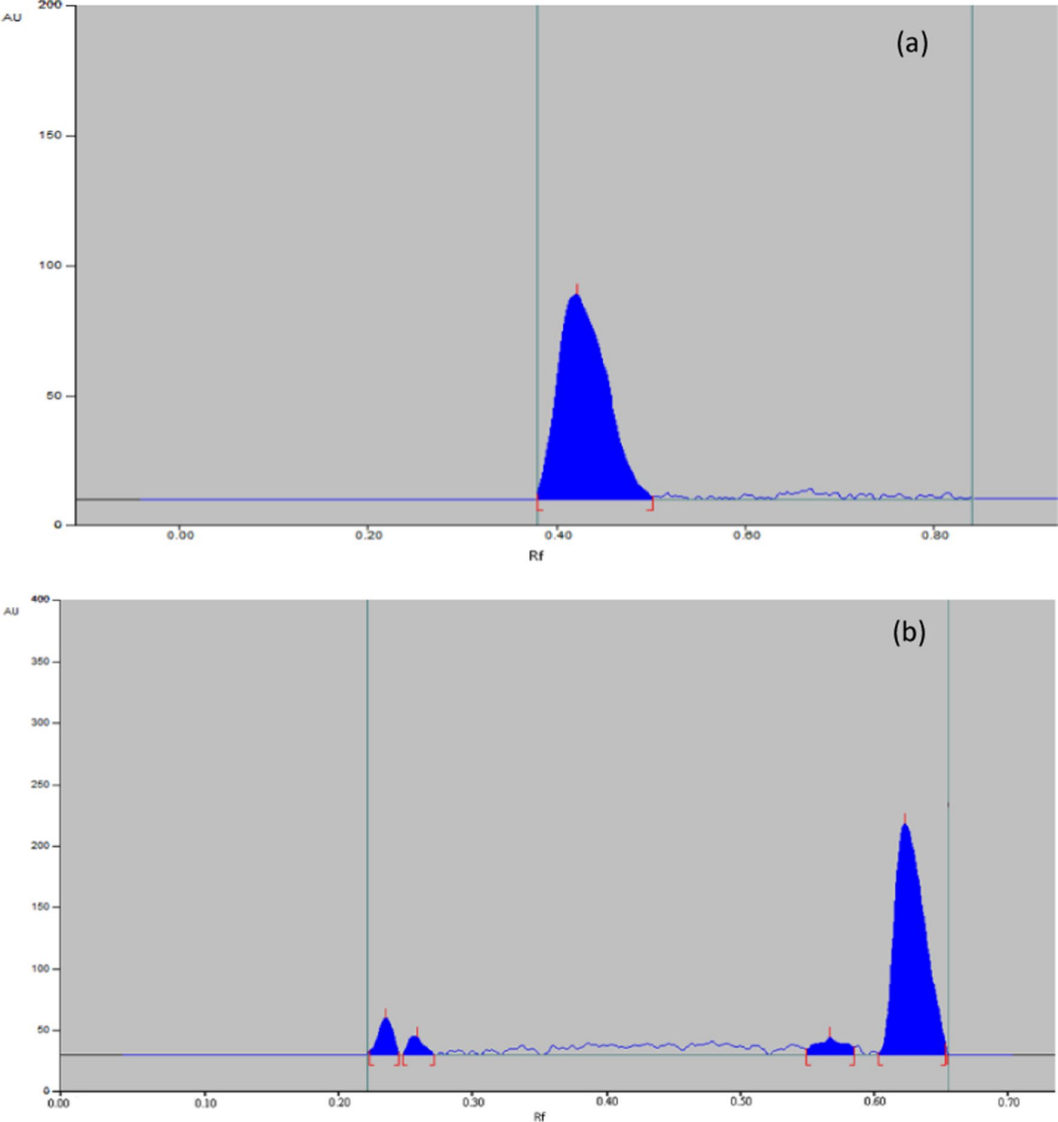
Representative chromatogram obtained the following exposure of MIR (**a**) and TAM (**b**) in their pharmaceutical dosage form to oxidative degradation

Both MIR and TAM were found to be liable to oxidative degradation. 75% of MIR was recovered with no additional peak, as shown in Fig. 9a, while 72% of TAM has recovered with additional peaks, as shown in Fig. 9b.

##### Thermal conditions

**Fig. 10:**
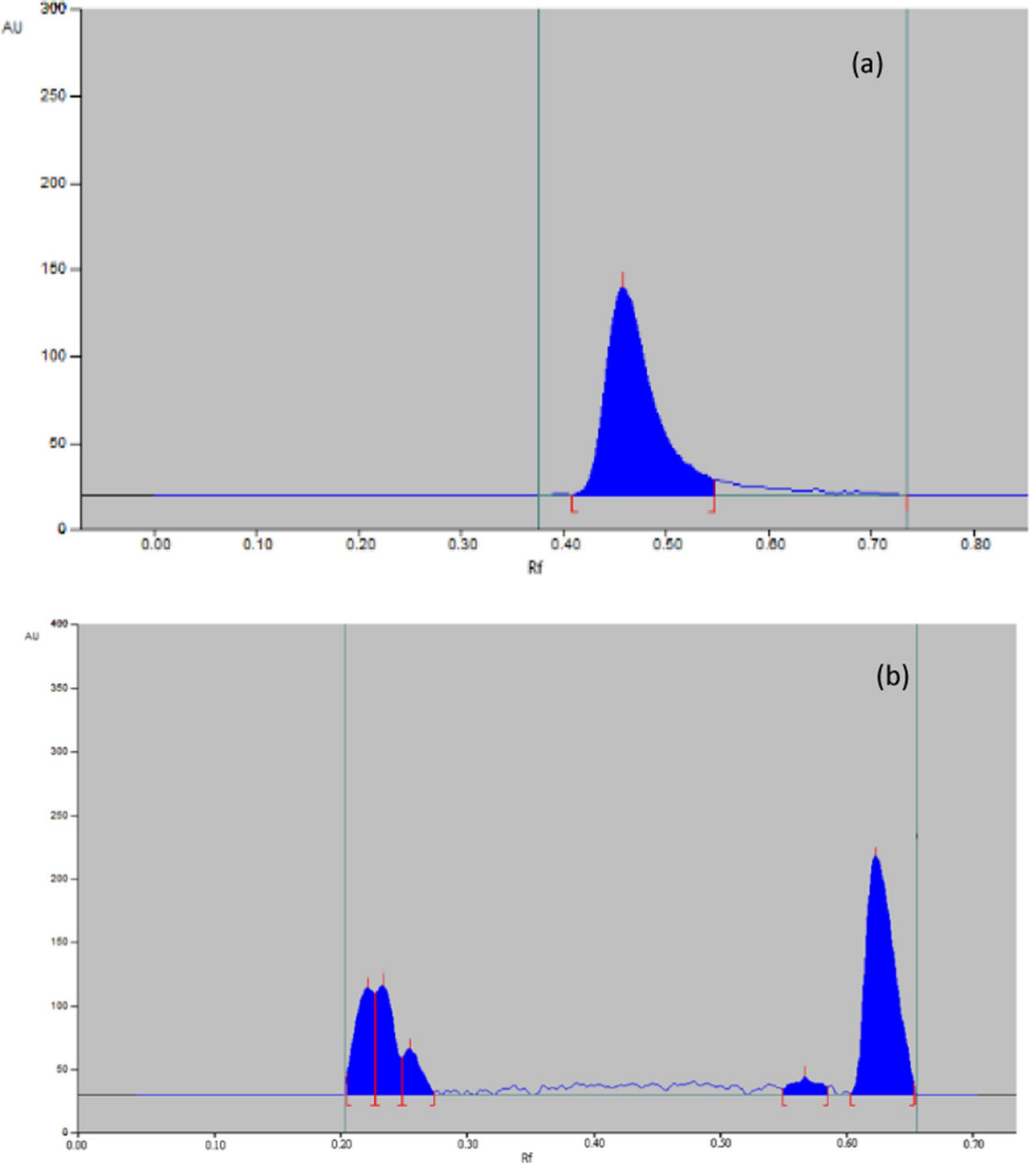
Representative chromatogram obtained the following exposure of MIR (**a**) and TAM (**b**) in their pharmaceutical dosage form to thermal degradation

After exposure of drugs to an oven at 80, Both MIR and TAM were found to be liable to thermal hydrolysis. 76% of MIR was recovered with no additional peak, as shown in Fig. 10a, while 82% of TAM was recovered with additional peaks as shown in Fig. 10b.

##### Photolytic conditions

**Fig. 11:**
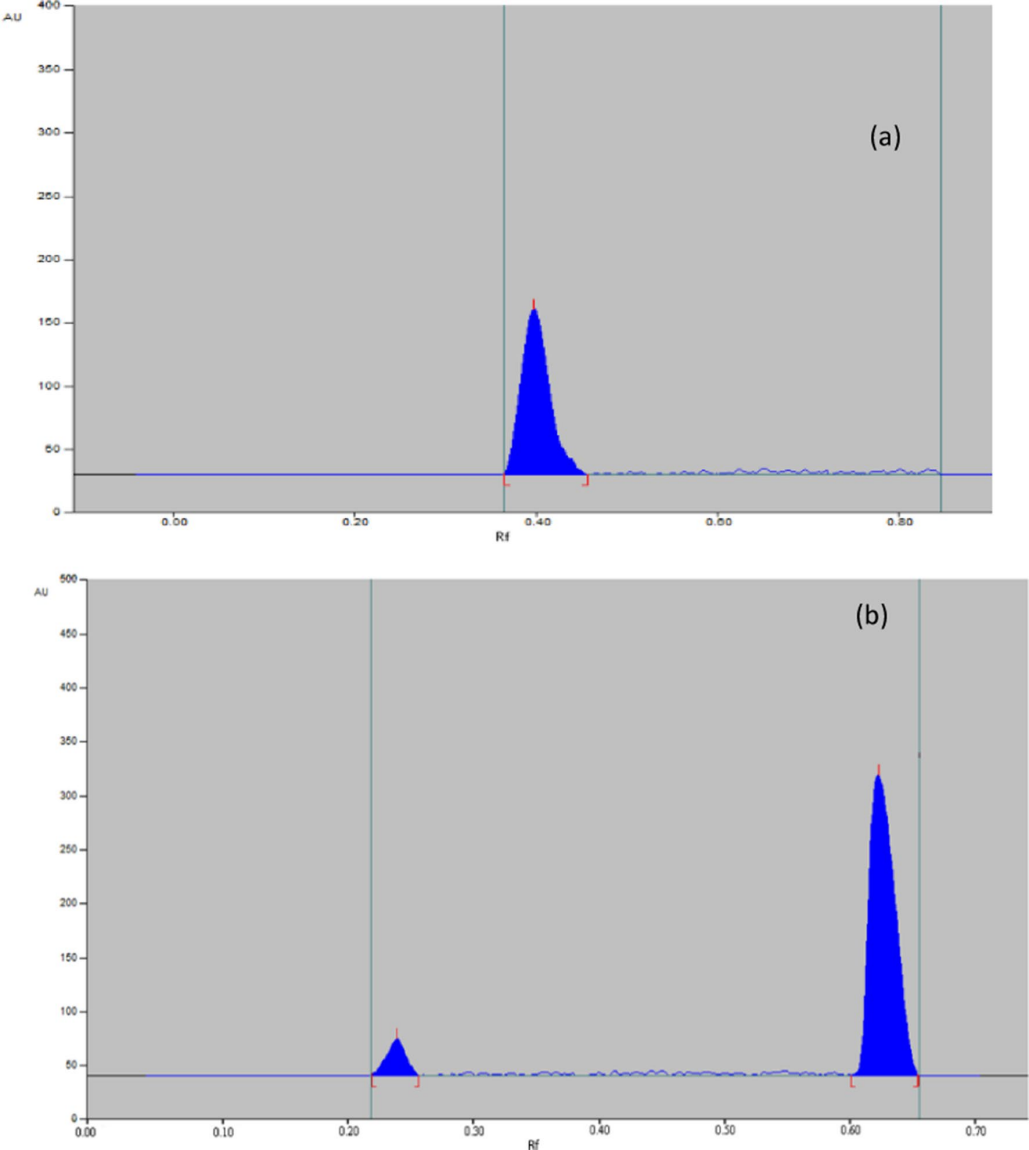
Representative chromatogram obtained the following exposure of MIR (**a**) and TAM (**b**) in their pharmaceutical dosage form to photolytic degradation

After exposure to sunlight for 24 h, MIR was found to be stable Fig. 11a. While 95% of TAM was recovered with an additional peak, as shown in Fig. 11b.

The results all degradation conditions are summarized in Table 8.

**Table 8:** Summary of the results of the forced degradation study of MIR and TAM under various conditions

Forced degradation conditions	% Recovered		Rf of degradation products	
	MIR	TAM	MIR	TAM
Alkaline	90	63	Not detected	0.22, 0.27, 0.29, 0.32, 0.5, 0.53, 0.56
Acidic	88	64	Not detected	0.23
Oxidative	75	72	0.89	0.22, 0.25, 0.56
Thermal	76	82	Not detected	0.22, 0.25, 0.27
Photolytic	Stable	95	stable	0.23

Related to MIR, the result of the stress degradation study is matched with the previous published article. MIR showed degradation under acidic, basic, hydrolytic and oxidative stress conditions but stable in photolytic condition [11]. While TAM showed degradation under acidic, basic, hydrolytic, photolytic and oxidative stress conditions [29]

#### Greenness assessment

The greenness of the developed HPTLC technique was assessed using three distinct green analytical chemistry metrics; Analytical eco-scale, GAPI, and AGREE. The goal of an analytical eco-scale metric is to quantify a method's green parameters. It is based on the concept of penalty points (PPs). Points for reagent toxicity, waste generation, and instrument energy consumption are removed from 100, and the number of residual points determines the result: > 75 represents excellent green analysis, > 50 represents acceptable green analysis, <50 represents inadequate green analysis. GAPI is another metric, and it may be used to evaluate the greenness of a whole analytical approach, from sample collection to final determination. The GAPI tool employs a pictogram to assess the greenness of each step of an analytical technique, with three color levels ranging from green to yellow to red. AGREE is the last metric. The AGREE metric's input criteria are based on the 12 SIGNIFICANCE principles and can be weighted differently, allowing for flexibility. Each of the 12 input variables is scaled from 0 to 1. The ultimate evaluation result is the sum of the assessment results for each principle. The output is a clock-like graph with the total score and color representation in the center. Software can be used to conduct the evaluation, which includes an automatically created graph and a report.

The greenness of the developed technique is shown in Table 9. The developed method has a score of 80 PP for analytical eco-scale compared to 77 PP for the reference methods of MIR and TAM. The difference in penalty points between the proposed and reference methods related to the volume of solvent used in HPTLC is less than that in HPLC. AGREE assessment shows that the proposed method is superior to the reported ones with 0.71 instead of 0.65 in the reported ones, the difference depends on many factor related to the CAS number of each solvent, the volume of solvent used, number of analyte in each run, toxicity of solvent to human and environment. According to Table 9, when evaluating using the GAPI metric, the proposed method indicated 5 yellow, 8 green, and 2 red regions, whereas the reference method indicated 5 green, 6 yellow, and 4 red regions which is related to toxicity and the volume of solvent used. Therefore, the proposed approach has a low ecological impact according to the above-mentioned greenness assessment metrics compared with the reported methods.

**Table 9:** Greenness assessment of the proposed HPTLC method

Methods (mobile phase)	GAPI	ECO-scale		AGREE
Proposed method (methanol-ethyl acetate-ammonia (3:7:0.1, v/v))		Reagents	16	
		Occupational hazard	0	
		Instrument	0	
		Waste	4	
		Total score	80	
HPLC for mirabegon [16] (acetonitrile: phosphate buffer (45:55, v/v))		Reagents	16	
		Occupational hazard	0	
		Instrument	1	
		Waste	6	
		Total score	77	
HPLC for tamsulosin [46] (acetonitrile: phosphate buffer (40:60, v/v))		Reagents	16	
		Occupational hazard	0	
		Instrument	1	
		Waste	6	
		Total score	77	

## Discussion

Recently, MIR is added to TAM in treating overactive bladder in men with benign prostatic hypertrophy as it improves overactive bladder symptoms and decreases micturition frequency [60]. This necessitates the development of a simple, specific technique for determining TAM and MIR in pure and pharmaceutical dosage form. The synergistic effect of co-adminstration of two cited drugs inspired us for development of selective, sensitive, and accurate method for simultaneous quantitation of MIR and TAM. The method was extended for determination of the active drugs together with degradation product.

Nowadays, chromatographic methods became the analytical methods of choice for qualitative and quantitative pharmaceutical analysis as

- Numerous samples could be determined simultaneously using a few amounts of mobile phase making this method more affordable and time-saving
- No prior extraction steps are required compared to HPLC.
- The developed plates could be determined numerous times if needed by storing them at appropriate conditions.
- No restrictions for the usage of the solvents as mobile phase or diluents because it is an open system.

In this work trials were done to develop HPTLC method which was able to separate and quantify the cited drugs and its degradation product in short analysis time with high sensitivity and selectivity. Also, efforts were attempted to use less hazardous solvents. Analytical eco scale, GAPI and AGREE were used to assess the greenness of the suggested method. Several trials were done to use greenness desirable solvents to separate all the studied components and their degradants.

## Conclusion

A green, rapid, selective, and economic HPTLC method using silica gel plate f254 was developed to simultaneously determine MIR and TAM in pure and laboratory prepared binary mixture. The greenness of the developed method was assessed using analytical eco-scale, GAPI, and AGREE metrics. The proposed method is green and economical with no need for a large amount of mobile phase and less time. The developed HPTLC method could also be used for routine analysis and stability studies of both drugs without any interference with degradation products.

## Data Availability

The data that support the findings of this study are available from the corresponding author upon request.
